# Expression and Differential Responsiveness of Central Nervous System Glial Cell Populations to the Acute Phase Protein Serum Amyloid A

**DOI:** 10.1038/s41598-017-12529-7

**Published:** 2017-09-22

**Authors:** Massimo Barbierato, Mila Borri, Laura Facci, Morena Zusso, Stephen D. Skaper, Pietro Giusti

**Affiliations:** Department of Pharmaceutical and Pharmacological Sciences, University of Padua, 35131 Padua, Italy

## Abstract

Acute-phase response is a systemic reaction to environmental/inflammatory insults and involves hepatic production of acute-phase proteins, including serum amyloid A (SAA). Extrahepatically, SAA immunoreactivity is found in axonal myelin sheaths of cortex in Alzheimer’s disease and multiple sclerosis (MS), although its cellular origin is unclear. We examined the responses of cultured rat cortical astrocytes, microglia and oligodendrocyte precursor cells (OPCs) to master pro-inflammatory cytokine tumour necrosis factor (TNF)-α and lipopolysaccaride (LPS). TNF-α time-dependently increased *Saa1* (but not *Saa3*) mRNA expression in purified microglia, enriched astrocytes, and OPCs (as did LPS for microglia and astrocytes). Astrocytes depleted of microglia were markedly less responsive to TNF-α and LPS, even after re-addition of microglia. Microglia and enriched astrocytes showed complementary *Saa1* expression profiles following TNF-α or LPS challenge, being higher in microglia with TNF-α and higher in astrocytes with LPS. Recombinant human apo-SAA stimulated production of both inflammatory mediators and its own mRNA in microglia and enriched, but not microglia-depleted astrocytes. Co-ultramicronized palmitoylethanolamide/luteolin, an established anti-inflammatory/ neuroprotective agent, reduced *Saa1* expression in OPCs subjected to TNF-α treatment. These last data, together with past findings suggest that co-ultramicronized palmitoylethanolamide/luteolin may be a novel approach in the treatment of inflammatory demyelinating disorders like MS.

## Introduction

Inflammation is a physiological process that assumes deleterious consequences if left unchecked^1^. Chronic inflammation underlies the development of pathologies both outside (e.g. rheumatoid arthritis, Crohn’s disease) and within (neuropathic pain, traumatic brain injury, spinal cord injury, chronic neurodegenerative disorders) the nervous system (‘neuroinflammation’)^2^. Neuroinflammation contributes importantly to the pathogenesis of chronic pain and neuropathic pain^3,4^, chronic neurodegenerative diseases^5,6^, neuropsychiatric illness^7,8^, autism spectrum disorder^9,10^, and probably even temporal lobe epilepsy^11^.

Inflammation involves the production of numerous mediators including cytokines, chemokines, reactive oxygen species, and acute phase proteins that are responsible for the accompanying physiological and metabolic changes. C-reactive protein, complement proteins and serum amyloid A protein (SAA) are some of the principal acute phase proteins, that are mainly produced in the liver and released into the systemic circulation in response to inflammation^12,13^. SAA is the generic name of a family of proteins with 103–104 amino acids that share high levels of sequence homology but are encoded by different genes^14^. Humans possess four SAA genes (SAA1, SAA2, SAA3 and SAA4) mapped in a 150-kb region of chromosome 11p15.1^15,16^. Mice also have 4 Saa genes, whose protein products are highly homologous to their human counterparts^14^. Inducible expression is characteristic of all acute-phase SAAs including SAA1 and SAA2. In addition, *Saa3* in mice is also an inducible SAA.^12^. SAA1 protein secreted by hepatocytes and released into the circulation is tightly bound to high-density lipoprotein (HDL) particles. Pro-inflammatory cytokines such as interleukin-1β (IL-1β), IL-6 and tumour necrosis factor-α (TNF-α), and glucocorticoids may play important roles in hepatic (the main source) expression of SAA1 and SAA2 during the acute-phase response^17^.

Extra-hepatic expression of SAA has also been reported^18^. Central nervous system (CNS) disorders are characterized by both central activation of innate immunity and activation of a potent peripheral acute phase response that influences central inflammation and contributes to poor outcome^19^. For example, intraperitoneal injection of lipopolysaccharide (LPS) in Syrian hamster brain resulted in *Saa* mRNA expression^20^. SAA might play a role in the inflammatory processes occurring in Alzheimer’s disease (AD) and the autoimmune demyelinating disease multiple sclerosis (MS). Although not detectable in normal brain, SAA protein has been described in AD brain, along with SAA gene expression in brain tumours and in brain tissue from MS patients^21^. SAA concentration was much higher in cerebrospinal fluid of AD subjects than in normal controls^22^, and SAA immunoreactivity co-localized with amyloid β-peptide deposits in AD brain^23^. Induction of a systemic acute phase response in SAA transgenic mice enhanced amyloid β-peptide deposition^24^. Intense immunohistochemical staining of SAA in brains of patients with neurologically confirmed AD and MS in comparison to an unaffected region and non-AD/MS brains has been reported, with the major site of SAA staining in both diseases being the myelin sheaths of axons in affected cortex^25^.

The above studies imply a role for SAA in inflammation-associated neuropathologies, but leave unanswered important questions, such as the cellular origin(s) of SAA in the CNS and the response of these cells to a disease-relevant inflammatory stimulus. The present investigation was designed to compare the response of CNS glia, namely astrocytes, microglia and oligodendrocytes to treatment with TNF-α in terms of *Saa* isoform gene expression, alongside an established pro-inflammatory stimulus, namely LPS. Our study also examined a role for astrocyte-microglial interaction in their responses to TNF-α. The pathophysiology of a variety of neurological disorders, including MS is associated with TNF-α^26,27^, a master pro-inflammatory product of activated microglia and peripheral macrophages implicated in the pathogenesis of CNS demyelination. Increased TNF-α in spinal cords coincides with neuropathic pain in rats undergoing experimental autoimmune encephalomyelitis^28^, and transgenic expression of TNF-α within the CNS leads to demyelinating disease^29,30^. Lastly, we examined whether co-ultramicronized palmitoylethanolamide/luteolin (co-ultraPEALut), given its anti-inflammatory and neuroprotective actions, would affect SAA expression in the above settings.

## Results

### TNF-α and LPS differentially up-regulate *Saa* gene expression in cultured cortical microglia and astrocytes

**Figure 1:**
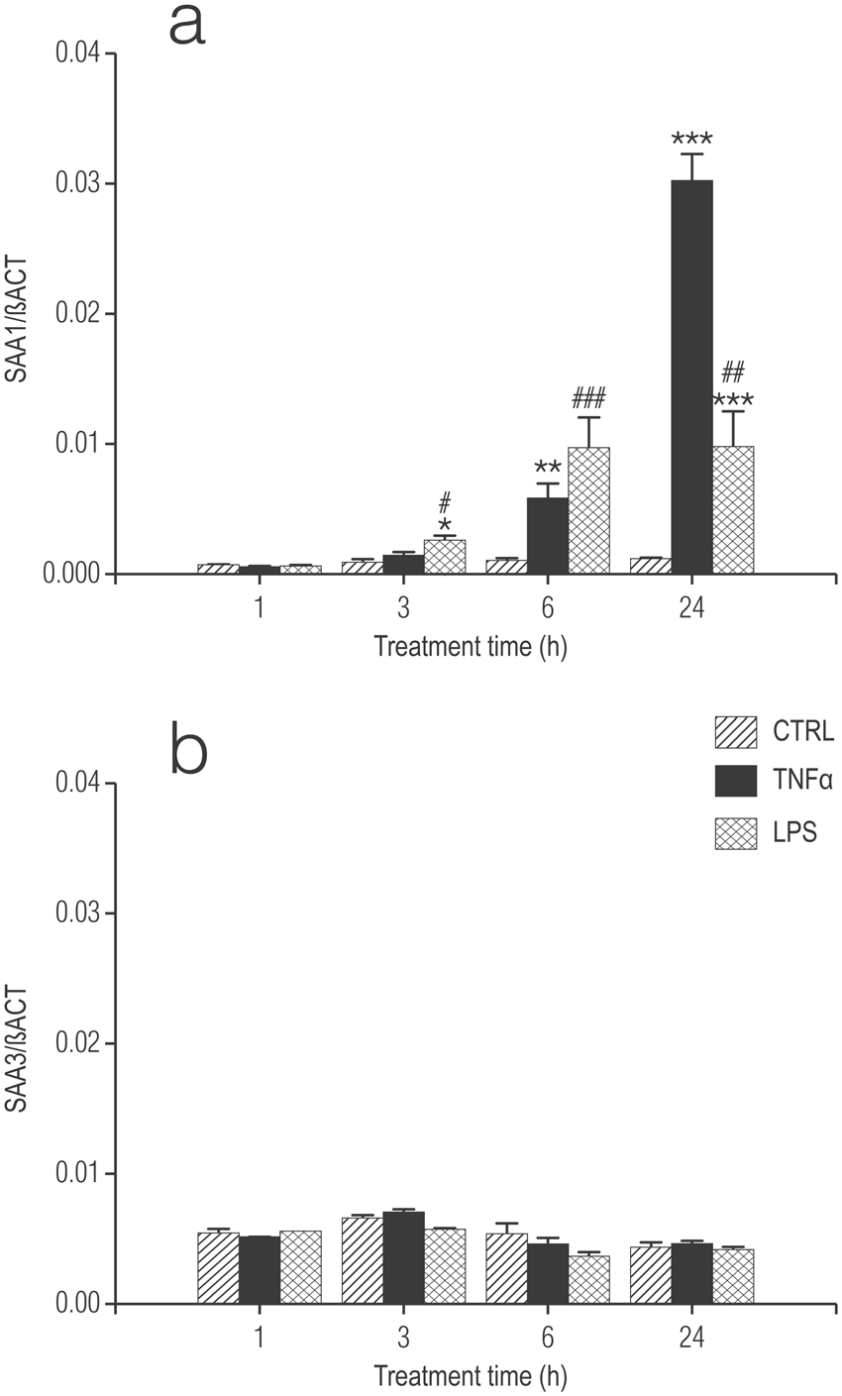
Treatment of rat cortical microglia with TNF-α or LPS up-regulates, in a time-dependent manner mRNA for *Saa1* but not *Saa3*. (**a**) SAA1; (**b**) SAA3. Cultures of microglia were treated the day after plating (DMEM + 0.5% FCS) with 10 ng/ml TNF-α or 100 ng/ml LPS and processed 1, 3, 6 and 24 h later for qRT-PCR, as detailed in Methods. Data are presented as relative expression level (normalized with respect to β-actin (βACT)) at each time point for the control (untreated) cultures and are means ± s.e.m. n = 6. *p < 0.05 *vs* control (CTRL); **p < 0.01 *vs* control; ***p < 0.001 *vs* control. ^#^ *p* < 0.05 *vs* control and TNF-α; ^##^ *p* < 0.01 *vs* control and TNF-α; ^###^ *p* < 0.001 *vs* control.

SAA mediates cytokine production by a variety of cell types, including macrophages, keratinocytes, monocytes, macrophages, neutrophils, THP-1 cells, U937 cells, HMC-1 mast cells, synoviocytes, endothelial cells, synovial fibroblasts, and chondrocytes^31^. However, its expression in defined CNS glial cell populations has not been well-documented. To explore this question, purified microglia were treated with TNF-α (10 ng/ml) (in line with concentrations used for microglia^32^ and epithelial cells^33^ or LPS (100 ng/ml). LPS is the major constituent of the outer wall of gram-negative bacteria and ligand for Toll-like receptor 4 (TLR4) and a well-established model for induction of an inflammatory response^34^. At different times over a 24-h period microglia were processed for qRT-PCR, and mRNA expression for the different *Saa* isoforms evaluated. TNF-α and LPS each caused a time-dependent rise in *Saa1* gene expression, being significantly greater compared to control at 6 and 24 h (Fig. 1a). At 24 h the effect of TNF-α was more robust than that of LPS. TNF-α treatment did not lead to any consistent changes in *Saa3* expression (Fig. 1b). *Saa1* remained elevated up to 6 days with either TNF-α or LPS challenge, but significantly higher with TNF-α (Supplementary Fig. S1a). The very low expression of *Saa4* mRNA rendered impractical a reliable measure of this isoform (data not shown). Microglia responded to LPS with a time-dependent increase in IL-1β in the culture medium (78.4 ± 3.0, 275.2 ± 19.4 and 514.8 ± 19.8 pg/ml, respectively, at 3, 6 and 24 h; values are mean ± s.e.m., n = 3); however, TNF-α treatment failed to elicit release of detectable amounts of this cytokine. Basal release of IL-1β was below the assay’s limit of detection.

**Figure 2:**
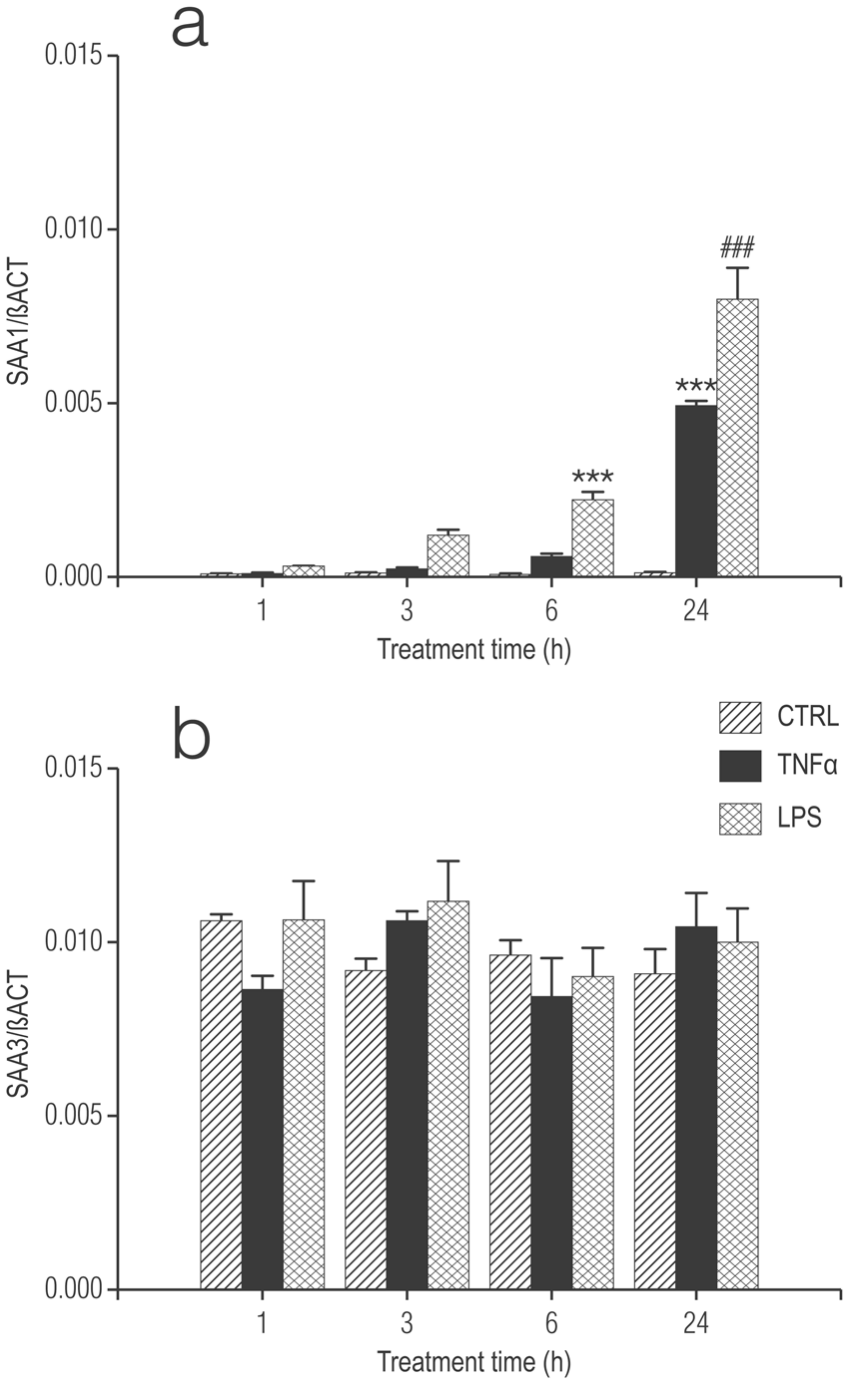
Treatment of enriched rat cortical astrocytes with TNF-α or LPS up-regulates, in a time-dependent manner mRNA for *Saa1* but not *Saa3*. (**a**) SAA1; (**b**) SAA3. Cultures were treated the day after plating (DMEM + 0.5% FCS) with 10 ng/ml TNF-α or 100 ng/ml LPS and processed 1, 3, 6 and 24 h later for qRT-PCR, as detailed in Methods. Data are presented as relative expression level (normalized with respect to β-actin (βACT)) at each time point for the control (untreated) cultures and are means ± s.e.m. n = 3. ***p < 0.001 *vs* control; ^###^ *p* < 0.001 *vs* control and TNF-α.

Enriched cortical astrocytes (<5% contaminating microglia; see Methods) incubated with TNF-α or LPS displayed also a significant and time-dependent rise in *Saa1* gene expression over 24 h, with the effect of LPS being greater than that of TNF-α (Fig. 2a). Neither TNF-α nor LPS affected *Saa3* gene expression (Fig. 2b). As with microglia, very low levels of *Saa4* made its measurement impractical. At longer times, and in contrast to microglia, the relative expression of *Saa1* gene continued to rise until at least 6 days (longest time examined) in TNF-α-treated astrocyte cultures and were significantly higher than for LPS-treated cells at this time (Supplementary Fig. S1b) – as seen for microglia. These data suggest that microglia display distinct responses to TNF-α and LPS as a function of the presence of astrocytes.

TNF-α (10 ng/ml) increased *Saa1* gene expression in oligodendrocytes precursor cells (OPCs) in a time-dependent fashion, being significantly greater than control at 6 and 24 h (Supplementary Fig. S2a). There were no statistically significant changes in *Saa3* gene expression (Supplementary Fig. S2b), even though expression of *Saa3* mRNA in control cultures was higher than for *Saa1*. Expression of *Saa4* mRNA was borderline detectable (data not shown). TNF-α, at the concentration used was not toxic to OPCs in the present experiments, as noted by others^35^. Effects of LPS were not examined in OPCs, as these cells do not express the LPS receptor TLR4^36,37^.

### Leu-Leu-OMe (L-LME) treatment of enriched astrocytes prevents induction of *Saa1* mRNA in response to TNF-α and LPS: effect of microglia re-addition

**Figure 3:**
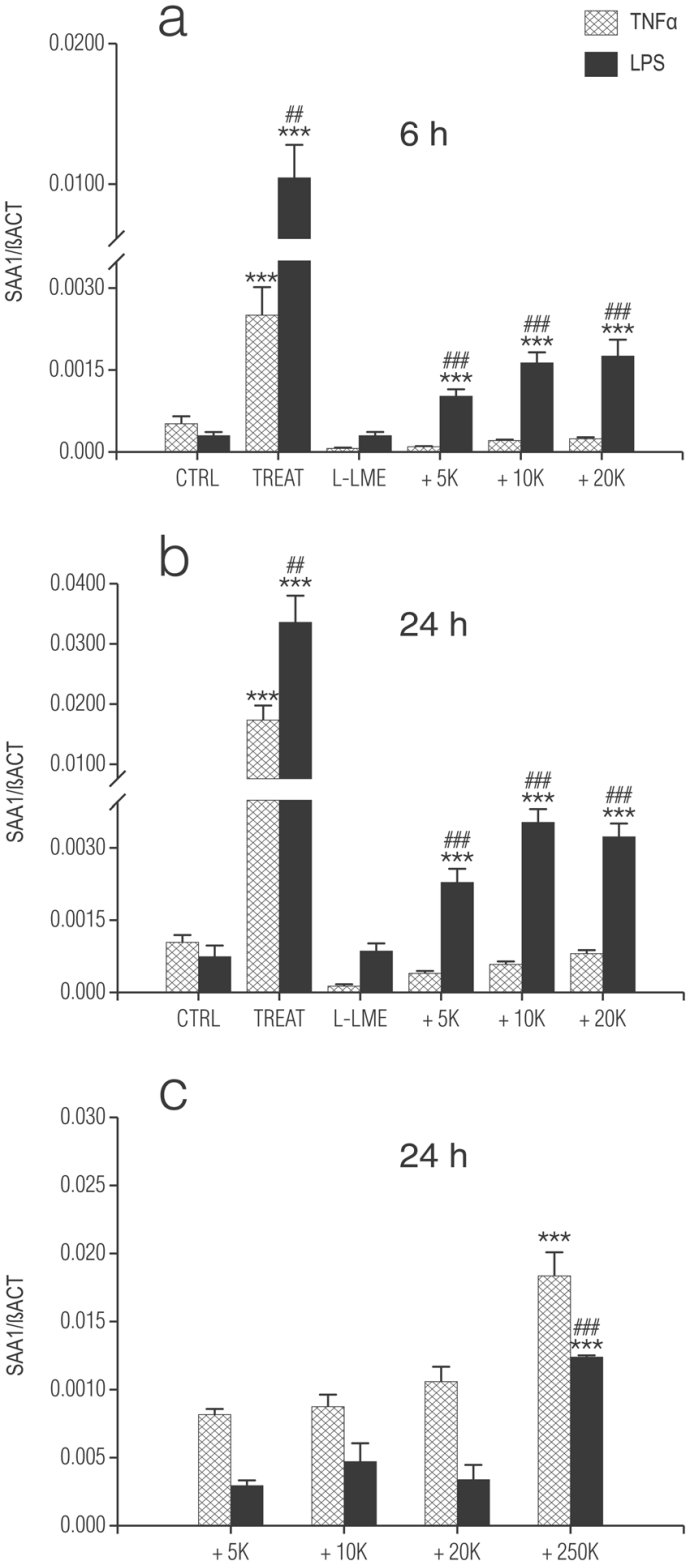
Addition of microglia to L-leucyl-L-leucine methyl ester (L-LME)-treated astrocytes fails to fully restore TNF-α and LPS-induced increases in *Saa1* mRNA. Enriched cortical astrocytes (250,000 per 24-well) were treated with 50 mM L-LME for 60 min, and returned to fresh culture medium for 24 h. After this time (medium change to DMEM + 0.5% FCS) purified cortical microglia were added, at the numbers indicated on the horizontal axis (+5 K, + 10 K, + 20 K, + 250 K) to the astrocyte cultures and incubation continued for a further 24 h. Cultures were then challenged with either 10 ng/ml TNF-α or 100 ng/ml LPS and cells processed for *Saa1* mRNA expression by qRT-PCR 6 h (**a**) and 24 h (**b**) later. Data are presented as relative expression level (normalized with respect to β-actin (βACT)) at each time point and are means ± s.e.m. n = 3. *p < 0.05 vs control (CTRL); **p < 0.01 vs control; ***p < 0.001 vs control. ^##^p < 0.01 vs TNF-α; ^###^p < 0.001 vs TNF-α. Similar results were obtained in a second experiment. (**c**) The same numbers of microglia were cultured in a parallel 24-well plate and subjected to the same treatments as above and then processed for *Saa1* mRNA expression by qRT-PCR at 24 h. For comparison, samples plated with 250,000 microglia per well were also analyzed. Data are presented as relative expression level (normalized with respect to β-actin (βACT)) and are means ± s.e.m. n = 3. ***p < 0.001 vs all other cell densities; ^###^p < 0.001 vs TNF-α.

Rodent primary astrocyte cell cultures prepared by standard protocols, including those used here generally contain variable, small percentages (up to 5%) of contaminating microglia^38^. Inflammatory mediator output from enriched astrocytes is dependent on the presence of residual microglia^39–43^. To interrogate the role of microglia in our cultures of enriched astrocytes, the lysosomotropic agent L-LME^41^ was employed to eradicate any remaining microglia^41,42,44–47^. Monolayers of enriched astrocytes were treated by a 60-min exposure to 50 mM-LME followed 24 h later by a 6-h or 24-h challenge with either TNF-α or LPS (Fig. 3a and b, respectively). Under these conditions L-LME treatment largely eliminated the responsiveness of enriched astrocytes to both stimuli. Under these conditions L-LME is not toxic to astrocytes^40–42,46^.

CNS microglia respond to an inflammatory challenge more robustly in the presence of astrocytes^41,42^. To determine if this is the case also for *Saa1* mRNA induction, purified microglia were re-introduced into cultures of L-LME-treated astrocytes. After allowing one day for attachment, cultures were challenged with either 10 ng/ml TNF-α or 100 ng/ml LPS and then processed for qRT-PCR 6 h (Fig. 3a) and 24 h (Fig. 3b) later. Relative expression of *Saa1* mRNA was significantly higher upon stimulation by LPS as compared to TNF-α (see also Fig. 2 and Supplementary Fig. S1b). L-LME treatment markedly diminished astrocyte responsiveness to the two stimuli at both time points. Assuming 4% impurity of astrocytes prior to L-LME (equivalent to 10,000 microglia for an astrocyte plating density of 250,000 cells per 24-well), 5,000, 10,000 and 20,000 microglia were added to the nominally microglia-free astrocytes. Incubation of such microglia/astrocyte ‘co-cultures’ with either TNF-α or LPS, although displaying significantly greater relative expression of the *Saa1* gene compared to L-LME-treated astrocytes alone did not reach values achieved with enriched astrocytes (Fig. 3a and b, respectively). In keeping with the behaviour of enriched astrocytes, *Saa1* gene expression was significantly higher at both 6 h and 24 h in reconstituted co-cultures challenged with LPS in comparison to TNF-α (again, see Supplementary Fig. 1b). In contrast, the same numbers of microglia cultured alone were more responsive to TNF-α as compared to LPS at 24 h (Fig. 3c) (see also Fig. 1a and Supplementary Fig. 1a).

Removal of residual microglia from enriched astrocytes effectively neutralized their ability to release IL-1β into the culture medium in response to LPS. Reintroduction of microglia (5,000–20,000) to nominally microglia-free astrocyte cultures did not restore LPS responsiveness, nor did the same numbers of microglia alone elaborate measureable quantities of IL-1β upon incubation with LPS. Increasing the number of microglia to 250,000, while producing quantifiable amounts of IL-1β in response to stimulation by LPS failed to reach values obtained with enriched astrocytes (data not shown).

### SAA stimulates production of inflammatory mediators as well as its own mRNA in microglia and in enriched, but not microglia-depleted astrocytes

SAA stimulation of cells results in transcriptional activation leading to increased levels of pro-inflammatory cytokines like IL-1β, TNF-α^48^ and nitric oxide (NO)^49^. These pro-inflammatory cytokines (IL-1β, IL-6, TNF-α) play important roles in hepatic expression of SAA1 and SAA2 during the acute-phase response^17^. Further, genetic deletion of the *Il-1β* gene leads to an impaired acute-phase inflammatory response in mice^50^, while deletion of the *Il-6* gene results in a compromised acute-phase response to tissue injury^51^.

**Figure 4:**
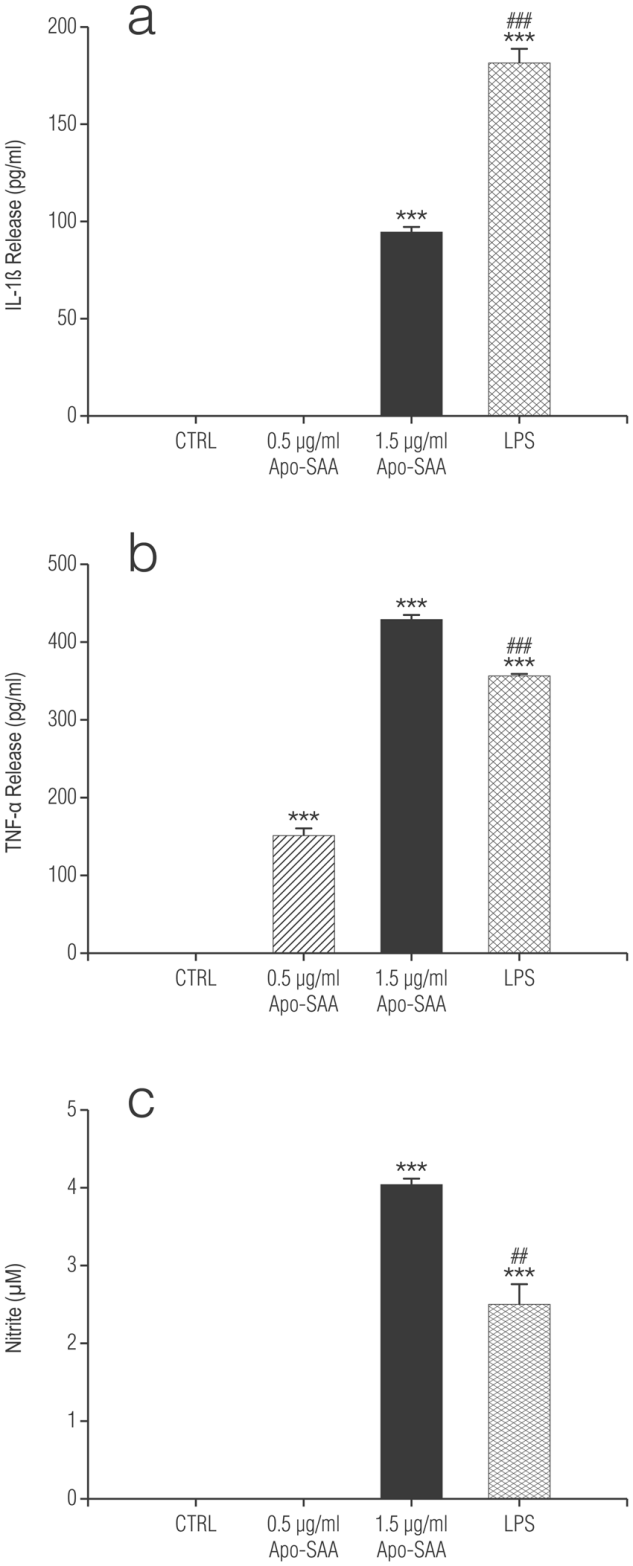
Recombinant human Apo-SAA or LPS treatment of rat cortical enriched astrocytes for 24 h increases the production of IL-1β (**a**), TNF-α (**b**) and NO (**c**). Cultures were treated the day after plating (DMEM + 0.5% FCS) with 0.5 or 1.5 μg/ml recombinant human Apo-SAA (Apo-SAA) or 100 ng/ml LPS and culture medium collected 24 h later for measurement of TNF-α, IL-1β by ELISA and NO by Griess reaction, as detailed in Methods. Data are expressed as means ± s.e.m. n = 3. ***p < 0.001 *vs* control (CTRL). ^##^p < 0.01 vs 1.5 µg/ml Apo-SAA; ^###^p < 0.001 vs 1.5 µg/ml Apo-SAA.

**Figure 5:**
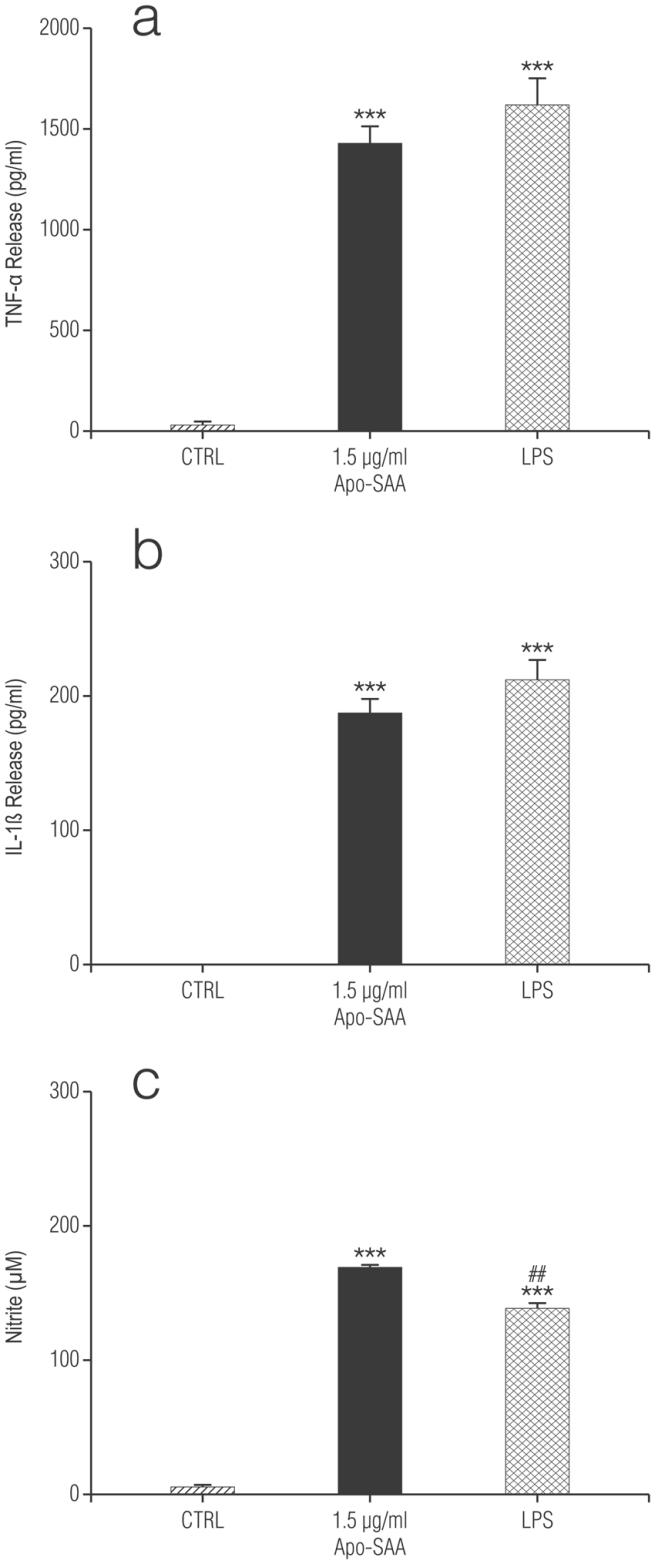
Recombinant human Apo-SAA or LPS treatment of purified rat cortical microglia for 24 h increases the production of TNF-α (**a**), IL-1β (**b**), and NO (**c**). Cultures were treated the day after plating (DMEM + 0.5% FCS) with 1.5 μg/ml recombinant human Apo-SAA (Apo-SAA) or 100 ng/ml LPS and culture medium collected 24 h later for measurement of TNF-α, IL-1β by ELISA and NO by Griess reaction, as detailed in Methods. Data are expressed as means ± s.e.m. n = 3. ***p < 0.001 *vs* control (CTRL). ^##^p < 0.01 vs 1.5 µg/ml Apo-SAA.

Treatment of enriched cortical astrocytes with increasing concentrations of recombinant human Apo-SAA (consensus SAA molecule corresponding to human Apo-SAA1α, except for the presence of an N-terminal methionine, the substitution of asparagine for aspartic acid at position 60, and arginine for histidine at position 71) for 24 h stimulated the production of IL-1β, TNF-α and NO (Fig. 4a, b and c, respectively). LPS, as expected, was also efficacious. In contrast, astrocytes depleted of microglia by treatment with L-LME were unresponsive (data not shown). Likewise, incubation of purified cortical microglia with Apo-SAA for 24 h induced the production of TNF-α, IL-1β and NO (Fig. 5a, b and c, respectively), as did LPS.

**Figure 6:**
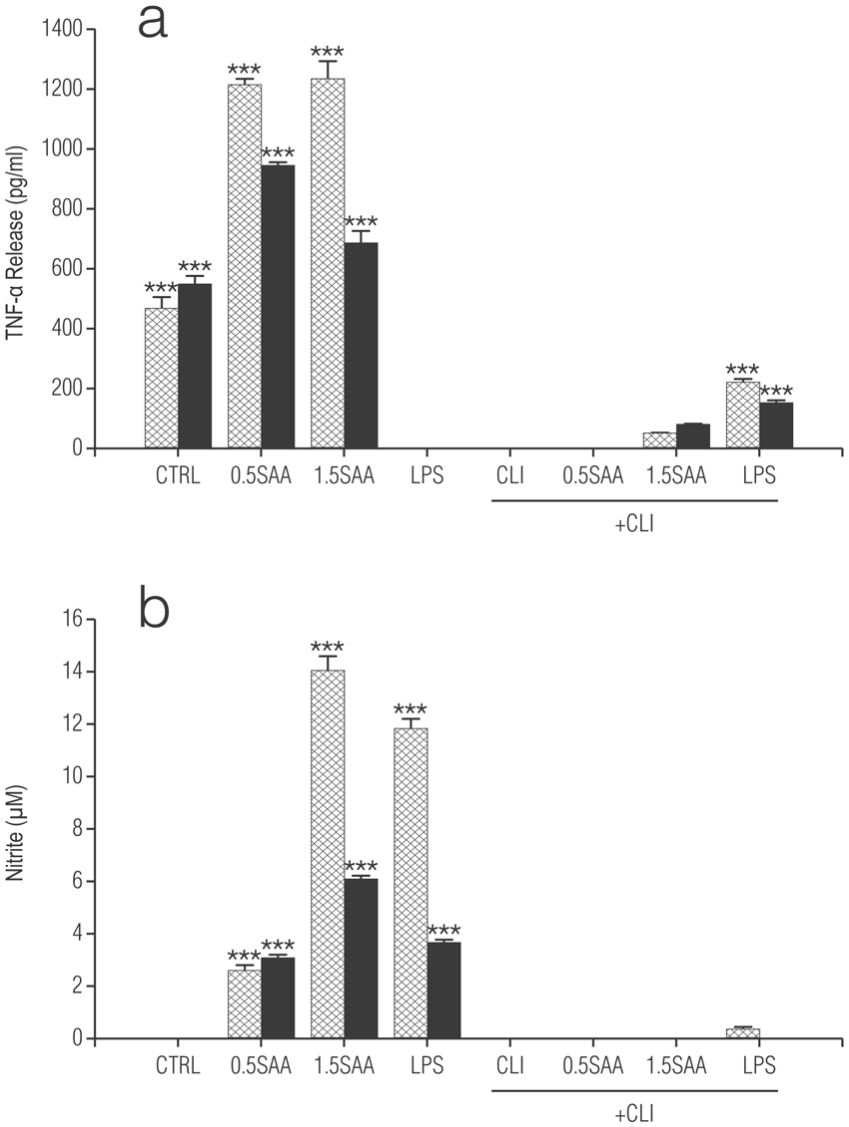
CLI-095 abolishes Apo-SAA- and LPS-stimulated production of TNF-α (**a**) and NO (**b**) by rat cortical enriched astrocytes (▓ ) and purified microglia (■) treated with recombinant human Apo-SAA or LPS for 24 h. Cultures were treated the day after plating (DMEM + 0.5% FCS) with 0.5 or 1.5 μg/ml recombinant human Apo-SAA ('SAA') or 100 ng/ml LPS, together with 0.5 μg/ml CLI-095 (‘CLI’). Culture medium was collected 24 h later for measurement of TNF-α (**a**) and NO (**b**) ELISA, as detailed in Methods. Data are expressed as means + s.e.m. n = 3. ***p < 0.001 vs control (CTRL). Similar results were obtained in a second experiment.

**Figure 7:**
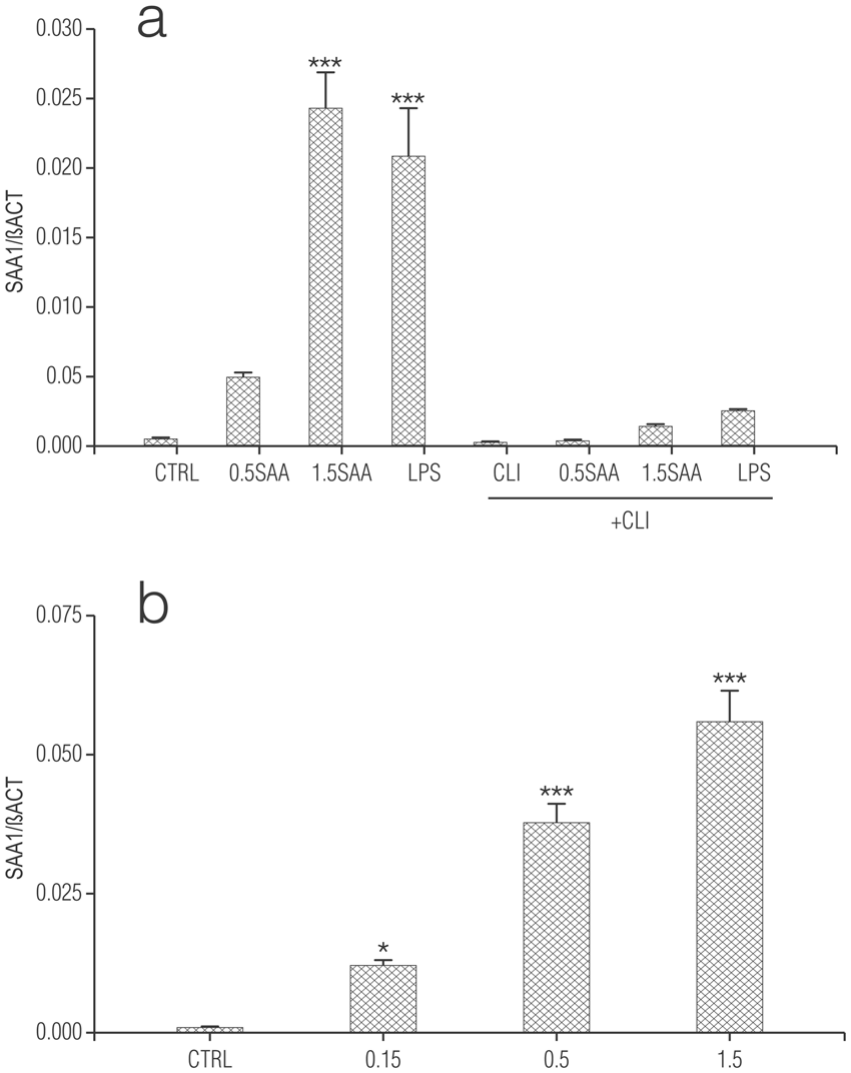
Treatment of rat cortical enriched astrocytes (**a**) and purified microglia (**b**) with recombinant human Apo-SAA or LPS for 24 h up-regulates expression of *Saa1* mRNA. Cultures were treated the day after plating (DMEM + 0.5% FCS) with the indicated concentrations (μg/ml) of recombinant human Apo-SAA ('SAA') (**a**,**b**) or 100 ng/ml LPS (**a**), together with 0.5 μg/ml CLI-095 (‘CLI’) (where indicated) (**a**) and processed 24 h later for qRT-PCR, as detailed in Methods. Data are presented as relative expression level (normalized with respect to β-actin (βACT)) and are means ± s.e.m. n = 3. *p < 0.05 *vs* control (CTRL); ***p < 0.001 *vs* CTRL. Similar results were obtained in a second experiment.

The actions of SAA are thought to depend on engagement of TLR4^49^. To explore this possibility in our cultures we used CLI-095, also known as TAK-242, a novel cyclohexene derivative that suppresses specifically TLR4 signalling, inhibiting the production of NO and pro-inflammatory cytokines^52^. It acts by blocking the signalling mediated by the intracellular domain of TLR4, but not the extracellular domain, and potently suppresses both ligand-dependent and -independent signalling of TLR4^53^. Treatment of rat cortical enriched astrocytes with CLI-095 (0.5 µg/ml) (in line with effective concentrations used for microglia^54^ and macrophages^52^) essentially abolished the release of both TNF-α (Fig. 6a) and NO (Fig. 6b) in cells stimulated with either recombinant human Apo-SAA or LPS, thus confirming that the effects of SAA and LPS are dependent on activation of TLR4. Polymyxin B, which prevents LPS binding to TLR4^55^ blocked the effects of LPS but not Apo-SAA in microglia (Supplementary Fig. S3). Treatment of enriched cortical astrocytes and purified microglia with recombinant human Apo-SAA for 24 h up-regulated also expression of *Saa1* mRNA itself (Fig. 7a and b, respectively) as did LPS (see also Fig. 3). CLI-095 (0.5 µg/ml) markedly reduced the up-regulation of *Saa1* gene in astrocytes stimulated with either recombinant human Apo-SAA or LPS (Fig. 7a), thus confirming that the effects of SAA and LPS are dependent on activation of TLR4. These data suggest possible autocrine/paracrine effects of SAA.

### Co-ultraPEALut limits TNF-α-induced *Saa1* gene expression in OPCs

**Figure 8:**
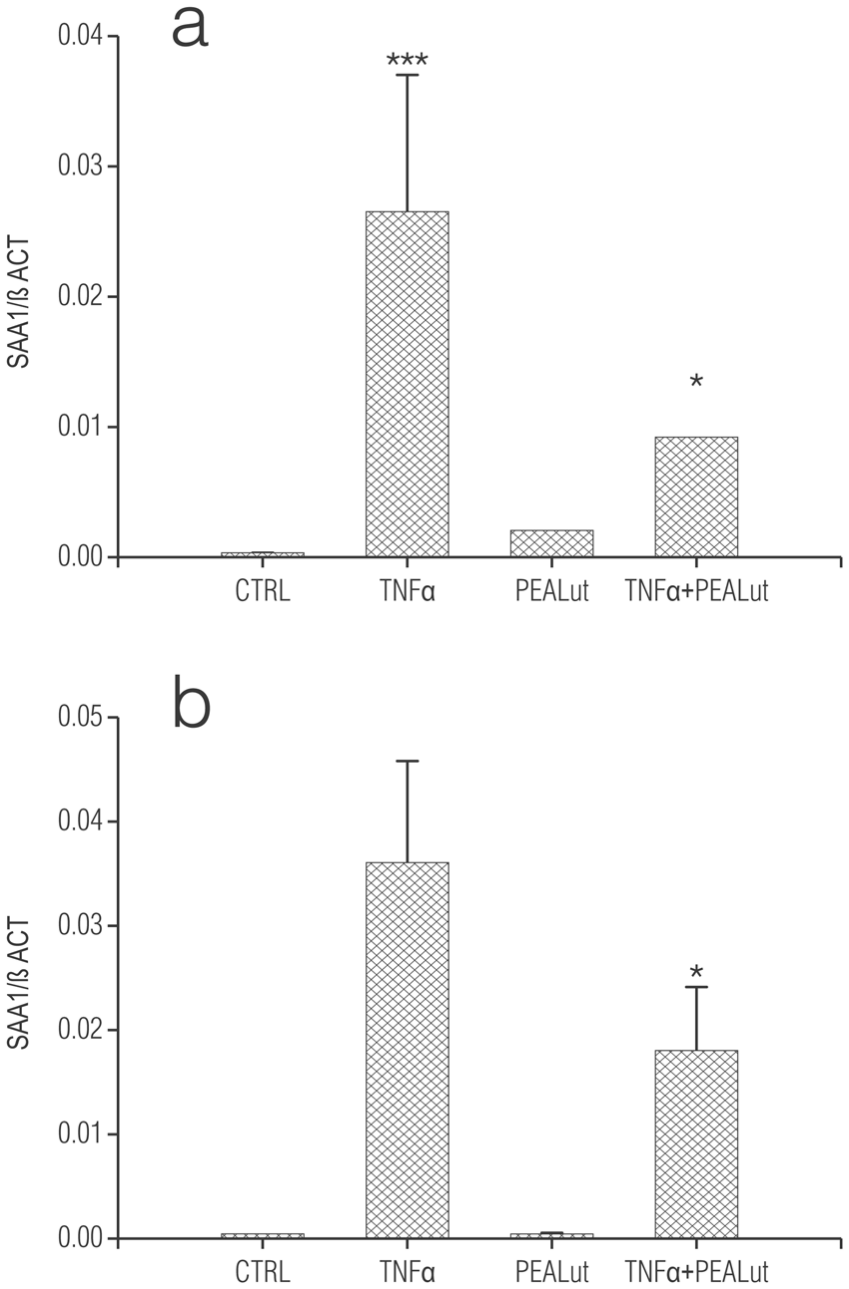
Co-ultramicronized PEA/luteolin (co-ultraPEALut) reduces *Saa1* gene expression in TNF-α-treated oligodendrocyte progenitor cells (OPCs) cultured in either Sato medium (**a**) or SFM (**b**). Isolated rat cortical OPCs were plated in Sato medium (without T3 and T4). One hour later this medium was replaced with either Sato medium or SFM. The following day, as indicated, co-ultraPEALut (‘PEALut’) was added (10 µM final) followed by TNF-α (final concentration: 10 ng/ml). Cells were processed 6 days later for qRT-PCR. Control (CTRL): culture medium with vehicle only. Data are means ± s.e.m. (**a**): n = 8 (3 experiments); (**b**): n = 12 (4 experiments). *p < 0.05 *vs* TNF-α. In a separate set of experiments, OPCs were treated as above and collected after 7 days. SFM: TNF-α (0.067 ± 0.010) and TNF-α + PEALut (0.039 ± 0.006) (n = 5, **p < 0.01); Sato medium: TNF-α (0.043 ± 0.016) and TNF-α + PEALut (0.018 ± 0.005) (n = 4).

N-palmitoylethanolamine, an endogenous fatty acid amide signalling molecule possesses analgesic, anti-inflammatory, and neuroprotective actions^56–58^. Recent studies show a co-ultramicronized composite of PEA and the flavonoid luteolin (co-ultraPEALut, 10:1 by mass) to be more efficacious that PEA alone in improving outcome in CNS injury models^58–63^. Co-ultraPEALut also promoted the biochemical and morphological development of OPCs cultured under conditions favouring either differentiation^64^ or proliferation^63^. To examine the possible effect of co-ultraPEALut on TNF-α-stimulated *Saa1* mRNA, OPCs were cultured in either Sato medium or SFM in the presence of TNF-α ± 10 µM co-ultraPEALut. The concentration of co-ultraPEALut was chosen based on our earlier studies examining the effects of this formulation on maturation of cultured OPCs^64^. Under these conditions, co-ultraPEALut significantly reduced the increase in *Saa1* mRNA relative expression induced by TNF-α after 6 days (Fig. 8a, b, respectively). No effects of co-ultraPEALut were observed in OPCs at shorter (1–2 days) incubation times or in enriched astrocytes after 6 days (data not shown).

## Discussion

Extensive evidence indicates that CNS disorders are primarily characterized by central activation of innate immunity, as well as activation of a potent peripheral acute phase response that influences central inflammation and leads to poor disease outcome^19^. The acute phase response plays a critical role in the innate immune response to tissue injury^65^. The present study was designed to investigate the cellular origin(s) of the acute phase protein SAA in the CNS, focusing on glia and their responses to disease-relevant inflammatory stimuli. Treatment of rat cortical purified microglia, enriched astrocytes, and OPCs with the pro-inflammatory cytokine TNF-α (as well as the first two cell populations with LPS) led to a time-dependent increase in mRNA expression of the *SAA* isoform *Saa1*. Isoform *Saa3* was unchanged, while *Saa4* (a constitutive form of SAA secreted by all cell types^17^) remained below the limits of detection. Depletion of residual microglia from enriched astrocytes markedly diminished the latter’s response to TNF-α and LPS, which could not be recovered by re-introduction of purified microglia. Microglia and enriched astrocytes showed distinct profiles when treated with TNF-α or LPS: *Saa1* relative expression in microglia was higher for TNF-α treatment as compared to LPS, with the opposite being true for enriched astrocytes or when microglia were re-added to microglia-depleted astrocytes. Recombinant human Apo-SAA stimulated production of not only inflammatory mediators but also its own mRNA in microglia and in enriched, but not microglia-depleted astrocytes. Further, co-ultraPEALut, an anti-inflammatory and neuroprotective agent, reduced *Saa1* gene expression in OPCs, but not enriched astrocytes, subjected to prolonged TNF-α treatment.

Our data are consistent with and extend earlier observations demonstrating that microglia responsiveness to a pro-inflammatory stimulus is enhanced when cultured with astrocytes^41,42^. In keeping with these prior reports, re-introduction of microglia to microglia-depleted astrocytes was insufficient to restore full responsiveness. In this context of microglia-astrocyte interaction it is interesting to note that relative expression of *Saa1* mRNA differed as a function of two factors: stimulation with TNF-α versus LPS and the response of microglia cultured alone or in the presence of astrocytes. The molecular basis for this interaction remains to be elucidated, although it does not appear to involve release of a soluble factor(s) by astrocytes^41,66^.

Rats do not develop amyloidosis and SAA is not an apoprotein of rat HDL. However, rats do have representatives of the SAA gene family; moreover, the pattern of genes expressed among tissues, and their induction by inflammatory agents, is similar to that of the related mouse genes^67^. This lends support for the importance of the SAA gene family in the response to injury by vertebrates, be they rodent or human.

SAA may activate TLR2^48^ and TLR4^49^, despite having little structural resemblance to the bacteria-derived ligands of either receptor. Commercially available SAA forms are recombinant proteins expressed in *Escherichia coli*, and the question rises whether contamination with LPS may account for some of its biological effects. We assessed this possibility by using CLI-095, a compound known to suppress specifically TLR4 signalling^52^ by blocking signalling mediated by the intracellular, but not extracellular, domain of TLR4^53^. Treatment of rat cortical enriched astrocytes and purified microglia with CLI-095 largely prevented the release of TNF-α and NO in cells stimulated with either recombinant human Apo-SAA or LPS, thus confirming that the effects of Apo-SAA and LPS are dependent solely on activation of TLR4. Polymyxin B, a cationic cyclic lipopeptide that binds stoichiometrically to the lipid A moiety of LPS and blocks its biological effects^55^ blocked the effects of LPS but not Apo-SAA. This result suggests that, at least in rat CNS glia under the present conditions recombinant human Apo-SAA mediates its effects through TLR4 but not TLR2, even though rat microglia^41,42^ and oligodendrocytes^68^ reportedly express functional TLR2.

SAA can be considered a “danger signal” that influences the inflammation process^69^, being produced when mammals sense potentially harmful environmental cues, including trauma, infection, tumour growth, surgery, and severe stress. Its low basal level and high inducibility are in keeping with danger signal molecules^70^. Given its chemotactic and cytokine-inducing activities, SAA may profoundly affect innate immunity. Its ability to activate TLR2 and TLR4 is of particular interest given that these pathogen-associated molecular patterns have important functions in innate immunity and inflammation and that their genetic variants are closely related to the disposition of several inflammatory and metabolic diseases featuring elevated SAA production^71^.

Inflammation is a key feature in the pathogenesis of many chronic peripheral and CNS pathologies. The *SAA* gene is reported to be expressed in brain of AD patients, individuals with brain tumours and MS, but not in brain from Pick’s disease, dementias with Lewy bodies or internal carotid infarct^21^. SAA has been documented immunohistochemically to co-localize with amyloid β-peptide deposits in AD brain^23^. SAA levels in cerebrospinal fluid (CSF) of AD subjects are much higher than in normal controls^22^, and generally within the range of the highest concentration used here. Induction of a systemic acute phase response in SAA transgenic mice reportedly enhanced deposition of amyloid β-peptide^24^. CSF-HDL is rich in apoE, and plays an important role as a ligand for lipoprotein receptors in CNS. Interestingly, addition of recombinant SAA to CSF dissociates apoE from CSF-HDL^22^. Because amyloid β-peptide binds to large CSF-HDL but not to apoE the authors postulated that inflammation in the CNS may impair amyloid clearance due to loss of apoE from CSF-HDL. Intriguingly, over-expression of SAA1 induces depressive-like behaviour in mice^72^. As SAA contains a cholesterol binding site near its amino terminus it is likely to have a high affinity for cholesterol-rich myelin. SAA can inhibit lipid synthesis in vascular smooth muscle cells^73^, and it is conceivable that SAA similarly inhibits lipid synthesis in oligodendrocytes and/or neurons. This inhibition may play a role in the white matter damage seen in both AD^74^ and MS^75^. The recent report that SAA induces inflammatory cytokine production in cultured cortical astrocytes (>95% purity) leaves open the question as to whether this is not a consequence of contaminating microglia^76^.

Building on our initial observations showing that a co-ultramicronized composite of the fatty acid amide PEA and the flavonoid luteolin promotes the morphological and molecular maturation of differentiating OPCs^64^ and improves the clinical score in myelin oligodendrocyte glycoprotein (MOG35-55)-induced experimental autoimmune encephalomyelitis in female C57BL/6 mice (a model often used as a chronic first-pass model of MS)^63^, we now demonstrate that co-ultraPEALut significantly limits the rise in *Saa1* gene expression in OPCs subjected to a 1-week exposure to TNF-α.

In conclusion, the present findings demonstrate that cortical oligodendrocytes, astrocytes and microglia respond to the pro-inflammatory cytokine TNF-α by an up-regulation of the acute phase protein SAA1, with the latter two cell types engaging in a crosstalk that reinforces the activation of microglia. Recombinant human Apo-SAA also stimulated up-regulation of its own gene, proposing autocrine/paracrine action and the existence of a feed-forward mechanism whereby release of TNF-α by activated microglia up-regulates SAA1 expression by nearby oligodendrocytes via type 1 TNF-α^77^ receptors (OPCs reportedly transcribe nearly undetectable levels of the type 2 receptor^77^) to further stimulate microglia, resulting in a ‘vicious cycle’. One cannot exclude the participation of type 2 receptors, however, as another study claimed that in normal adult rat brain oligodendrocytes express TNF‐type 2 but not type 1 receptor; after 3 days in culture, both types of receptors were expressed by mature oligodendrocytes^78^. However, it would be impractical (and rather beyond the scope of the present study) to evaluate TNF receptor subtype involvement in these experiments. In every case, these observations may have particular relevance in the pathophysiology of autoimmune demyelinating diseases, including MS, where TNF-α appears to play a prominent role^26–28,79^. Collectively, past and current data propose that co-ultraPEALut may be a novel approach in the treatment of inflammatory demyelinating disorders, as well as in other CNS pathologies classically viewed as primarily neuronal diseases but where myelin and oligodendrocyte loss are also relevant.

## Methods

### Materials

Tissue culture media, N2 supplement, antibiotics and fetal calf serum (FCS) were obtained from Life Technologies (San Giuliano Milanese, Italy); poly-D-lysine hydrobromide (mol wt 70,000–150,000), poly-L-lysine hydrobromide (mol wt 70,000–150,000), cytosine β-D-arabinoside, 3,3′,5-triiodo-L-thyronine, L-thyroxine, lipopolysaccharide (LPS; Ultra-Pure LPS-EB from E. coli 0111:B4 strain) and ethyl-(6 R)-6-(N-(2-chloro-4-fluorophenyl)sulfamoyl)cyclohex-1-ene-1-carboxylate (CLI-095 or TAK 242) were from InvivoGen (Cayla-Invivogen Europe, Toulouse, France), Griess reagent, papain, DNase I (bovine pancreas), trypsin inhibitor, L-LME, recombinant human fibroblast growth factor-2 (FGF2), recombinant rat TNF-α (T5944, cell culture tested, endotoxin level <0.1 ng/µg as determined by the Limulus amebocyte lysate method), recombinant human platelet-derived growth factor AA and all other biochemicals were purchased from Sigma-Aldrich (Milan, Italy) unless noted otherwise; recombinant human Apo-SAA (consensus SAA molecule corresponding to human Apo-SAA1α, except for the presence of an N-terminal methionine, the substitution of asparagine for aspartic acid at position 60, and arginine for histidine at position 71) from Peprotech (London, UK); Falcon tissue culture plasticware was purchased from BD Biosciences (SACCO srl, Cadorago (CO), Italy). Sterilin petri plastic dishes (10 cm Ø) were from Sarstedt (Verona, Italy). Co-ultramicronized PEA/luteolin (10:1 mass ratio) was kindly provided by Epitech S.p.A., Saccolongo (PD), Italy.

### Primary culture of microglia and astrocytes

Microglia were isolated from mixed glial cell cultures as previously described^80^. Experiments were performed in accordance with Italian Ministry of Health (art. 31, D.L. 26/2014) guidelines for the care and use of laboratory animals, and were approved by the Institutional Animal Care and Use Committee of the University of Padua (958/2016-PR). Briefly, cells dissociated from P1-P2 rat pup (strain: CD) cerebral cortices were plated in 75-cm^2^ poly-L-lysine-coated tissue culture flasks (1.5 brains per flask) and grown in high-glucose Dulbecco’s modified Eagle’s medium (DMEM) with 2 mM glutamine, 50 units/ml penicillin/50 μg/ml streptomycin, 50 µg/ml gentamycin and 10% FCS (glial cell growth medium). Culture medium was changed after 24 h. The cultures reached confluence by 7 days at which time microglia were recovered by shaking the flasks on an orbital shaker at 200 rpm for 1 h (37 °C). The attached cell monolayers were highly enriched in astrocytes (<5% microglia, as determined by flow cytometry using cell type-specific antibodies)^66^. The culture supernatant containing microglia was transferred to plastic Petri dishes (Sterilin) and incubated for 45 min at 37 °C (5% CO_2_, 95% air) to allow differential adhesion of microglia. The adherent microglial cells (>99% pure, as determined by flow cytometry using cell type-specific antibodies)^66^ were detached by mechanically scraping into glial cell growth medium and replated in this same medium, on poly-L-lysine-coated microwell culture plates or dishes. The flaskswere re-fed with fresh medium (12 ml/flask) and returned to the incubator for another 7 days. These will be used to collect OPCs (following section).

For experiments where microglia were added back to microglia-depleted astrocyte cultures, microglia on Sterilin dishes were maintained in growth medium for a further 2 days until harvest. In some cases astrocytes were depleted of residual microglia using a 60-min exposure (50 mM) to the lysosomotropic agent L-LME^46^, as described previously^41^. Culture medium was exchanged for fresh medium, andcells allowed to recover for 1 day in growth medium prior to experimentation.

### Primary culture of oligodendrocyte precursor cells

Flasks used for collection of microglia and astrocytes were subjected to a second cycle of rotary shaking (6 h); the culture supernatant was subsequently transferred to plastic Sterilin Petri dishes and incubated for 45 min at 37 °C (5% CO_2_/95% air) to allow differential adhesion of any remaining microglia. The final cell suspension (containing >96% oligodendrocytes^81^) was collected and centrifuged (200 *g*, 5 min). The resulting cell pellet was re-suspended in serum-free medium (DMEM containing 1x N2 supplement, 50 U/ml penicillin and 50 μg/ml streptomycin, 0.5% (v/v) FCS) at 75,000 or 150,000 cells per well in a 24-well plate coated with poly-D-lysine and left at room temperature for 30 min to allow for uniform cell attachment/distribution^82^, followed by 30 min at 37 °C (5% CO_2_/95% air). The medium was then changed to one of the following (1 ml/well): ‘differentiation’ medium [Sato medium (DMEM supplemented to contain 400 ng/ml 3,3′,5-triiodo-L-thyronine, 400 ng/ml L-thyroxine, 2 mM-glutamine, 50 U/ml penicillin and 50 μg/ml streptomycin, 1x N2 supplement) and 0.5% (v/v) FCS] for cultures plated at 150,000 cells/well; ‘proliferation’ medium (‘SFM’) (DMEM containing 1x N2 supplement, 20 nM hydrocortisone, 10 ng/ml D-biotin, 5 ng/ml FGF2, 5 ng/ml PDGF-AA, 0.1% (w/v) bovine serum albumin) for cultures plated at 75,000 cells/well. Cultures were maintained at 37 °C in a 5% CO_2_/95% air incubator. After 24 h cytosine β-D-arabinoside (10 μM; to inhibit growth of any residual astrocytes) was added to cultures in Sato medium.

### Culture treatments

Microglia and astrocytes were seeded in poly-L-lysine-coated 24-well plates at a density of 250,000 cells per well, using glial cell growth medium and allowed to adhere overnight. Twenty-four hours later cells were then incubated with TNF-α (10 ng/ml) or LPS (100 ng/ml) in either ‘proliferation’ medium or Sato medium for the times indicated in each experiment, then processed for qRT-PCR analysis. Culture supernatants were retained and stored at −20 °C for analysis of IL-1β, TNF-α and NO contents. OPCs, cultured in either SFM (75,000 cells/24-well) or Sato medium (150,000 cells/ml) were treated with TNF-α (10 ng/ml) either on the day of plating or the following day. After different times of incubation, as indicated in each experiment, cells were processed for qRT-PCR analysis.

### Preparation of co-ultramicronized PEA/luteolin solutions

Co-ultramicronized PEA/luteolin was prepared as a 5 mM stock solution in 10% (w/v) Pluronic F-68. Concentration was calculated based on the molecular weight of PEA (the co-ultramicronized PEA/luteolin composite contains PEA and luteolin in a 10:1 mass ratio). The co-ultramicronized PEA/luteolin solution was sonicated for 20 min in a Elmasonic S (Singen, Germany) sonicating water bath. The co-ultramicronized PEA/luteolin solution was then diluted into culture medium at 100x the desired final concentration, and added (10 µl/1 ml) directly to the cell cultures without exchange of medium. The concentration of Pluronic F-68 was maintained constant at 0.02%, and added to the control culture wells also. After 30 min incubation co-ultramicronized PEA/luteolin was added, as above.

### qRT-PCR

Total RNA was extracted from cells by TRIzol^®^ (Invitrogen), according to the manufacturer’s instructions. RT was performed with Superscript III reverse transcriptase (Invitrogen). The qRT-PCR reaction was performed as described previously^41^. Primer sequences are listed in Table 1. Amounts of each gene product were calculated using linear regression analysis from standard curves, demonstrating amplification efficiencies ranging from 90 to 100%. Dissociation curves were generated for each primer pair, showing single product amplification. Data are normalized to β-actin mRNA level.

**Table 1:** PCR primers used in this study.

Target	Direction	Sequence
β-ACT	F	5′-CCCCATTGAACACGGCATTGTCA-3′
	R	5′-ACCCTCATAGATGGGCACAGTGT-3′
IL-1β	F	5′-TGTGGCAGCTACCTATGTCT-3′
	R	5′-GGGAACATCACACACTAGCA-3′
SAA1	F	5′-ACACGGAGCAGAGGACTCAAG-3′
	R	5′-GGTCGAAAGTGGTTGGGGTC-3′
SAA3	F	5′-AGGCAGCTTCCCAAGTGTGA-3′
	R	5′-ACTTCAAACCACAGAAAACACGA-3′
SAA4	F	5′-TGGGACTTGTGCAGAGCCTATC-3′
	R	5′-CCTTTGTTGGGCCTCGAAGT-3′
TNF-α	F	5′-CATCTTCTCAAAACTCGAGTGACAA-3′
	R	5′-TGGGAGTAGATAAGGTACAGCCC-3′
Iba1	F	5′-AACTGGAGGCCTTCAAGACG-3′
	R	5′-AACCCCAAGTTTCTCCAGCA-3′

β-ACT, β-actin; SAA, serum amyloid A; IL-1β, intereukin-1β; iNOS, inducible nitric synthase; TNF-α, tumor necrosis factor α; Iba1, ionized calcium binding adaptor molecule 1. F, forward; R, reverse.

### Enzyme-linked immunosorbent assays (ELISA) for IL-1β and TNF-α

IL-1β and TNF-α contents of culture medium were analyzed using commercially available enzyme-linked immunosorbent assay (ELISA) kits according to the manufacturer’s instructions (Antigenix America, Huntington Station, NY, USA). Standards with known amounts of IL-1β and TNF-α were used to convert values into absolute concentrations of IL-1β and TNF-α in pg/ml.

### NO Assay

NO has a relatively short half-life. Hence, quantitative assessment of NO production has generally relied on the indirect measurement of its oxidized products, nitrite and nitrate, which are regarded as suitable markers of NO generation. Equal volumes of cell culture medium and Griess reagent (Sigma-Aldrich) were incubated for 15 min, and the amount of nitrite quantified using a standard curve of sodium nitrite at O.D. 540 nm.

### Statistics

Data are given as mean ± s.e.m. (standard error of the mean). Statistical analyses to determine group differences were performed either by two-sample equal variance Student’s *t* test, or by one-way analysis of variance, followed by Dunnett’s or Bonferroni’s post-hoc test for comparisons involving more than two data groups.

### Data Availability

The datasets generated during and/or analysed during the current study are available from the corresponding author on reasonable request.

## Electronic supplementary material

Supplementary Information
