# Supplementary material for: Expression and Differential Responsiveness of Central Nervous System Glial Cell Populations to the Acute Phase Protein Serum Amyloid A
Source: Sci Rep. 2017 Sep 22;7:12158. doi: 10.1038/s41598-017-12529-7 (PMC5610307; doi:10.1038/s41598-017-12529-7)
Supplement: Supplementary file 1 — Supplementary Information [file 41598_2017_12529_MOESM1_ESM.pdf]

Supplementary Information

Expression and Differential Responsiveness of Central nervous System Glial Cell Populations to the Acute Phase Protein Serum Amyloid A

Massimo Barbierato, Mila Borri, Laura Facci, Morena Zusso, Stephen D. Skaper and Pietro Giusti

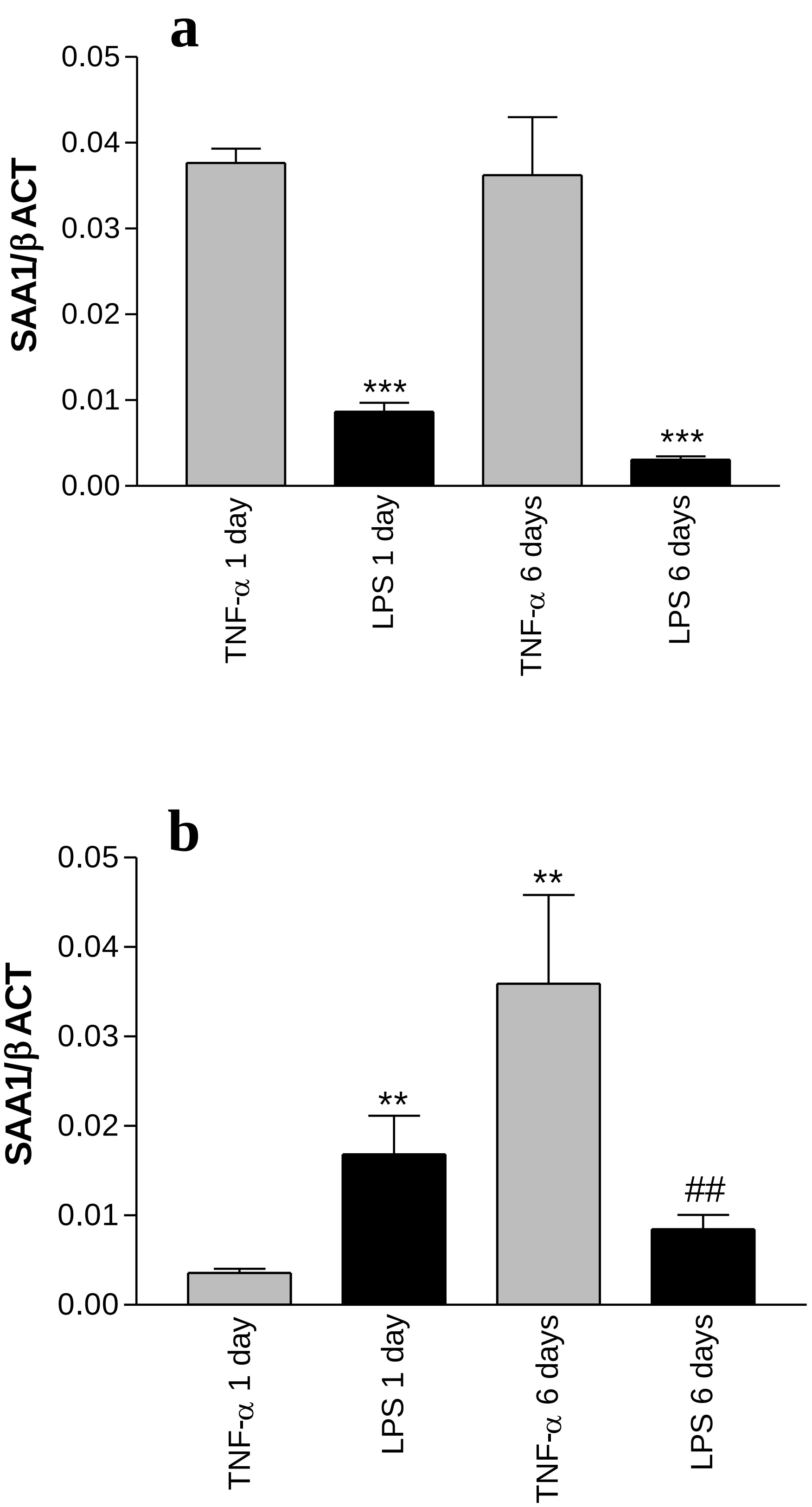
a
SAA1/β ACT
0.05
0.04
0.03
0.02
0.01
0.00
***
***
TNF-α 1 day
LPS 1 day
TNF-α 6 days
LPS 6 days
b
SAA1/β ACT
0.05
0.04
0.03
0.02
0.01
0.00
**
**
##
TNF-α 1 day
LPS 1 day
TNF-α 6 days
LPS 6 days

Supplementary Figure S1. Purified rat cortical microglia (**a**) and enriched astrocytes (**b**) display differential timings in expression of mRNA for *Saa1* in response to treatment with TNF-α or LPS. Cultures were treated the day after plating (DMEM + 0.5% FCS) with 10 ng/ml TNF-α or 100 ng/ml LPS and processed 1 and 6 days later for qRT-PCR, as detailed in Methods. Data are presented as relative expression level (normalized with respect to β-actin (βACT)) at each time point and are means + s.e.m. n=6 (n=3 for microglia at 6 days). (**a**) ***$p<0.001$ vs LPS at 1 day and 6 days, respectively. (**b**) **$p<0.$vs TNF-α at 1 day; $^{\#\#}p<0.01$ vs TNF-α at 6 days. Expression values for control (untreated cells have been omitted, owing to their very low levels (<0.001).

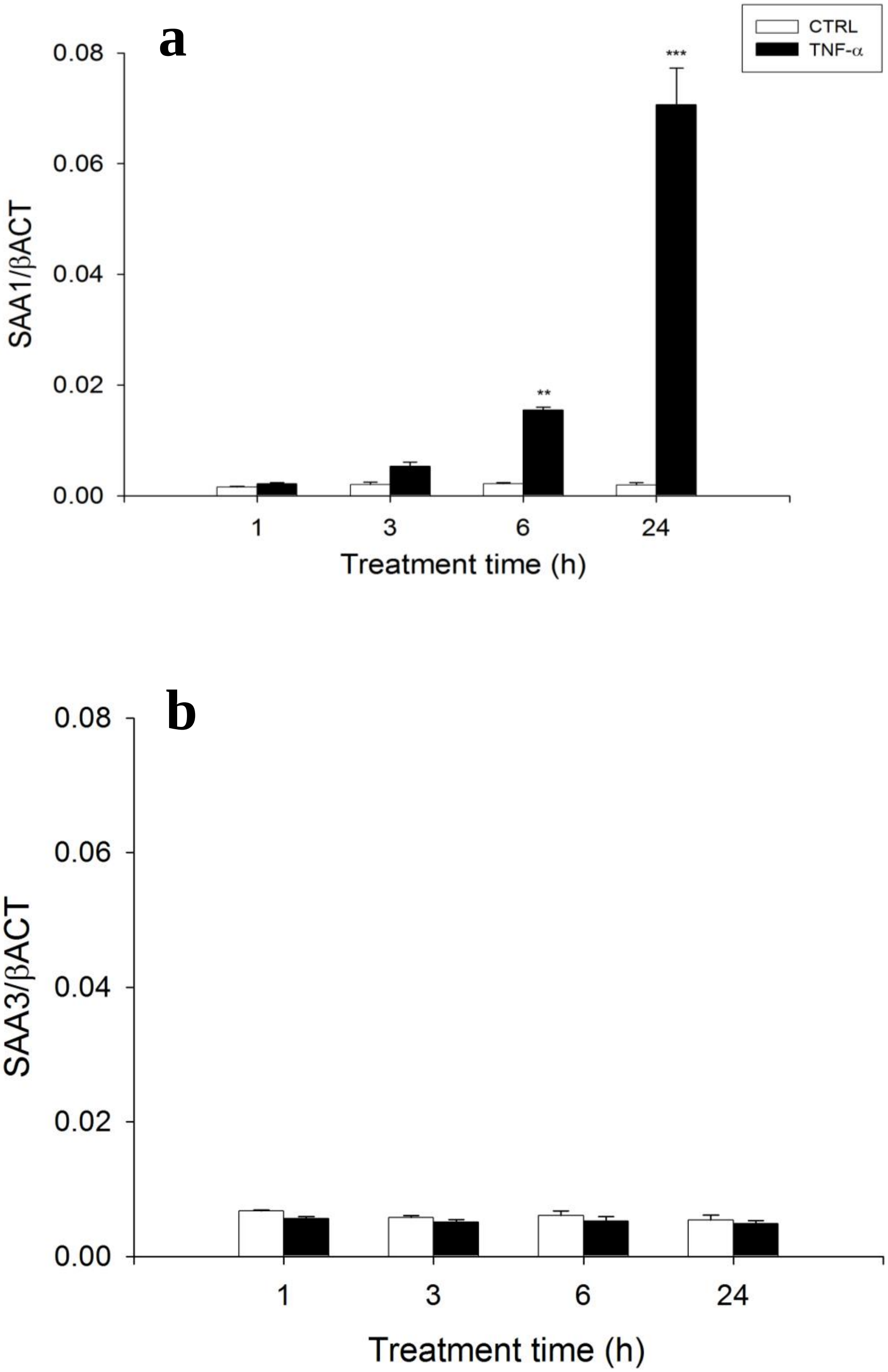
a
CTRL
TNF-α
SAA1/βACT
0.08
0.06
0.04
0.02
0.00
***
**
1
3
6
24
Treatment time (h)
b
SAA3/βACT
0.08
0.06
0.04
0.02
0.00
1
3
6
24
Treatment time (h)

Supplementary Figure S2. Treatment of rat cortical OPCs with TNF-α up-regulates, in a time-dependent manner mRNA for *Saa1* but not *Saa3*. (**a**) SAA1; (**b**) SAA3. Cultures of OPCs were treated the day after plating (Sato medium) with 10 ng/ml TNF-α and processed 1, 3, 6 and 24 h later for qRT-PCR, as detailed in Methods. Data are presented as relative expression level (normalized with respect to β-actin (βACT)) at each time point and are means + s.e.m. n=3. **p<0.01 vs control (CTRL); ***p<0.001 vs control.

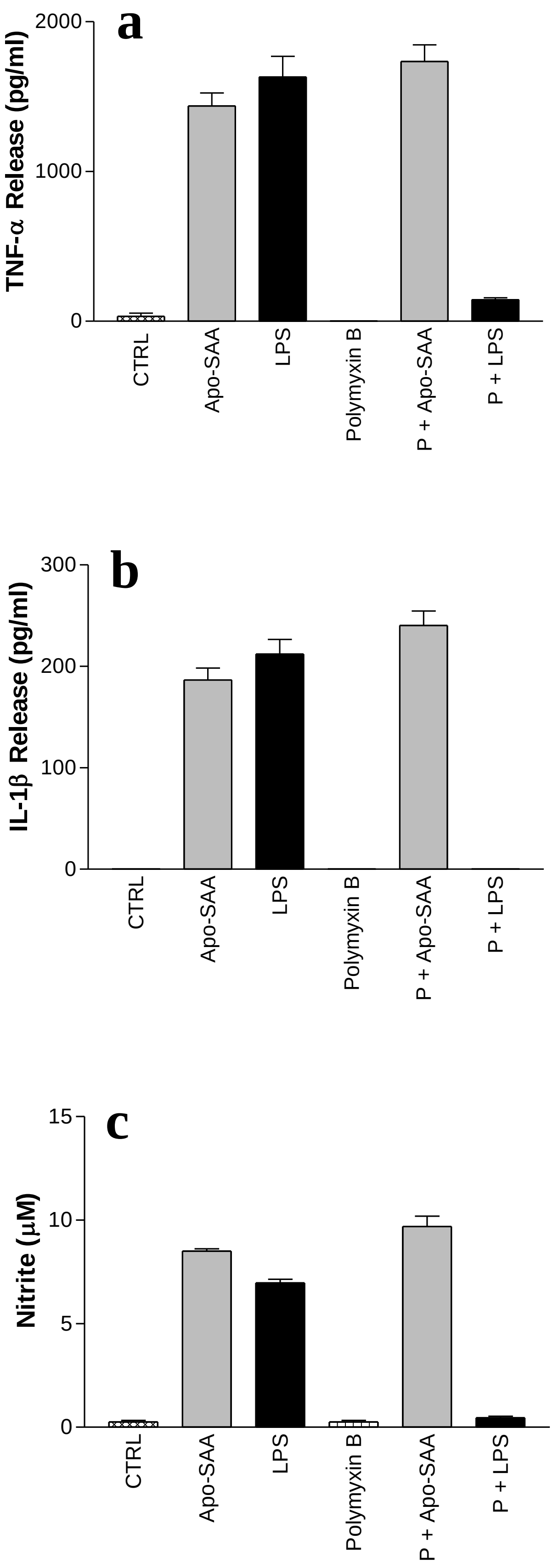
a
TNF-α Release (pg/ml)
2000
1000
0
CTRL
Apo-SAA
LPS
Polymyxin B
P + Apo-SAA
P + LPS
b
IL-1β Release (pg/ml)
300
200
100
0
CTRL
Apo-SAA
LPS
Polymyxin B
P + Apo-SAA
P + LPS
c
Nitrite (μM)
15
10
5
0
CTRL
Apo-SAA
LPS
Polymyxin B
P + Apo-SAA
P + LPS

Supplementary Figure S3. Polymyxin B blocks the effect of LPS, but not apo-SAA, on stimulation of inflammatory mediator release from rat cortical microglia. Cultures were treated the day after plating (DMEM + 0.5% FCS) with with polymyxin B (‘P’) (10 μg/ml) followed 30 min later by 1.5 μg/ml recombinant human Apo-SAA (Apo-SAA) or 100 ng/ml LPS and culture medium collected 24 h later for measurement of (**a**) TNF-α and (**b**) IL-1β by ELISA, or (**c**) NO by Griess reaction. Data are expressed as means ± s.e.m. n=3.
